# Low to No Effect: Application of tRNS During Two-Digit Addition

**DOI:** 10.3389/fnins.2018.00176

**Published:** 2018-04-05

**Authors:** Silke M. Bieck, Christina Artemenko, Korbinian Moeller, Elise Klein

**Affiliations:** ^1^LEAD Graduate School & Research Network, University of Tuebingen, Tuebingen, Germany; ^2^Leibniz-Institut für Wissensmedien, Tuebingen, Germany; ^3^Department of Psychology, University of Tuebingen, Tuebingen, Germany

**Keywords:** transcranial random noise stimulation, addition problems, two-digit addition, intraparietal sulcus, dorsolateral prefrontal cortex

## Abstract

Transcranial electric stimulation such as transcranial random noise stimulation (tRNS) and transcranial direct current stimulation (tDCS) have been used to investigate structure-function relationships in numerical cognition. Recently, tRNS was suggested to be more effective than tDCS. However, so far there is no evidence on the differential impact of tDCS and tRNS on numerical cognition using the same experimental paradigm. In the present study, we used a two-digit addition paradigm for which significant—albeit small—effects of tDCS were observed previously to evaluate the impact of parietal and frontal tRNS on specific numerical effects. While previous studies reported a modulation of numerical effects of this task through tDCS applied to parietal areas, we did not observe any effect of parietal tRNS on performance in two-digit addition. These findings suggest that tRNS seemed to influence concurrent mental arithmetic less than tDCS at least when applied over the IPS. These generally small to absent effects of tES on actual arithmetic performance in the current addition paradigm are in line with the results of a recent meta-analysis indicating that influences of tES may be more pronounced in training paradigms.

## Introduction

Numerical cognition in general and mental arithmetic in particular are important multi-dimensional competences for which neuroimaging studies suggest various brain regions to be involved (see Arsalidou and Taylor, 2011 for a meta-analysis on brain activation in mental arithmetic). As such, there is increasing interest in specifying the neurocognitive basis of numerical cognition. However, from neuroimaging studies alone it cannot be derived which brain structures are functionally relevant for numerical cognition because structures necessary for numerical cognition can hardly be separated from those that are just co-activated. One possible approach to investigate the functional relevance of specific cortex areas for cognitive processes in general and numerical cognition in particular is to externally manipulate the activation of these areas by applying transcranial electrical stimulation (tES) and then evaluate possible changes in behavior. The underlying idea is to modulate (i.e., either facilitate or impair) numerical processing by activating or inhibiting certain cognitive processes subserved by the respective stimulated areas. Typically, it is of specific interest whether or not numerical cognition can be improved by tES applied to brain areas assumed to be critically involved. In the present study, we describe the results of an experiment which followed-up studies by Klein et al. (2009, 2013), and Artemenko et al. (2015) systematically investigating the neural correlates of mental addition by using functional magnetic resonance imaging (fMRI) and tES, respectively. In the following, we will first introduce tES and recent evidence from its effects on numerical cognition, before we outline the specifics of the present study.

### Transcranial electric stimulation

tES has been suggested as a method which can enhance domain-general (involving attention, working memory, etc.) as well as domain-specific processes in numerical cognition (e.g., magnitude representation, place-value processing, etc., Cohen Kadosh et al., 2010; Schroeder et al., 2017) by stimulating the respective brain areas subserving these processes. At the moment, the most promising and most frequently used methods in this area are transcranial direct current stimulation (tDCS) and transcranial random noise stimulation (tRNS). However, these two stimulation methods have different operating modes.

During tDCS low-intensity constant current is applied (usually 0.5–2 mA; for an overview see Nitsche et al., 2008; Woods et al., 2016; Antal et al., 2017). tDCS is known to modulate cortical excitability. As a rule of thumb it can be assumed that anodal tDCS increases cortical excitability by elevating the neural resting membrane potentials closer to the activation threshold without directly triggering action potentials (Bikson et al., 2004). In contrast, cathodal tDCS is assumed to decrease excitability of the underlying brain tissue by lowering the resting membrane potential. In the majority of studies, anodal stimulation was found to improve human performance, while cathodal stimulation impaired human performance (for a review see Kuo and Nitsche, 2012; but see Jacobson et al., 2012; Li et al., 2015; Fertonani and Miniussi, 2017 for individual differences as well as the compexitiy and non-linearity of stimulation effects).

On the other hand, during tRNS randomly alternating current is applied by adding neural noise. The exact nature of the effective mechanisms are still unknown, but it is argued that the increase of background noise boosts the neural signal toward the activation threshold (e.g., Paulus, 2011; Cohen Kadosh, 2015). This phenomenon can be explained by stochastic resonance (Moss et al., 2004). tRNS was first introduced by Terney et al. (2008). The authors observed that motor cortex excitability of healthy subjects increased significantly while applying current with a random amplitude and in a high frequency range (100–640 Hz).

As regards the effectiveness of tDCS compared to tRNS, Moliadze et al. (2014) compared anodal tDCS, intermittent theta burst stimulation (iTBS) and tRNS on the motor cortex (i.e., M1) by evaluating motor evoked potentials (MEPs). Although all three stimulation methods significantly increased motor cortex excitability, tRNS showed the strongest and longest MEP increase compared to sham. However, so far, there is no direct comparison of the impact of tDCS and tRNS on arithmetic processing. Nevertheless, such a comparison for the case of numerical cognition would be interesting because Snowball et al. (2013) were able to show long-lasting tRNS effects even 6 months after stimulation in numerical cognition.

### tES in numerical cognition research

There are several studies that investigated the influence of tDCS as well as tRNS on arithmetic learning (e.g., Cohen Kadosh et al., 2010; Snowball et al., 2013) and arithmetic processing (e.g., Clemens et al., 2013; Rütsche et al., 2015). These studies suggest a possible beneficial effect of stimulation by enhancing numerical cognition in general. Nevertheless, most neurostimulation studies in basic arithmetic research have been conducted with tDCS only. So far, tRNS has primarily been successfully used in numerical intervention studies (Cappelletti et al., 2013; Snowball et al., 2013; Popescu et al., 2016; Looi et al., 2017; but see Pasqualotto, 2016 for an arithmetic processing study).

For instance, Snowball et al. (2013) applied tRNS over the dorsolateral prefrontal cortex (DLPFC) during calculation learning and drill learning. The authors showed that learning rates were elevated by prefrontal tRNS for both calculation and drill tasks. Moreover, even 6 months after the training took place the authors still found a benefit in arithmetic performance due to stimulation during the training. In another training study, Popescu et al. (2016) applied prefrontal tRNS during days 1–3 and parietal tRNS during days 4–5 when investigating its impact on arithmetic problem solving. This combination of stimulation protocols was observed to improve performance. Moreover, applying tRNS also successfully improved concurrent arithmetic processing. Pasqualotto (2016) found that while applying frontal or parietal tRNS, participants responded faster during a subtraction verification task, but not during a word classification task. Overall, participants were faster when they received frontal tRNS stimulation. These studies indicate that both, prefrontal as well as parietal placements resulted in effective performance increases using tRNS. However, for tRNS especially prefrontal application seemed to be more effective so far.

Nevertheless, tES research on arithmetic is heterogeneous as regards the processes investigated, the tasks employed as well as the stimulation protocols used (Schroeder et al., 2017). Therefore, it is difficult to directly compare the outcomes and implications of these studies. An exception is a line of research consistently using the same addition paradigm employing different stimulation techniques and protocols.

### A systematic approach on tES in numerical cognition research

**Figure 1:**
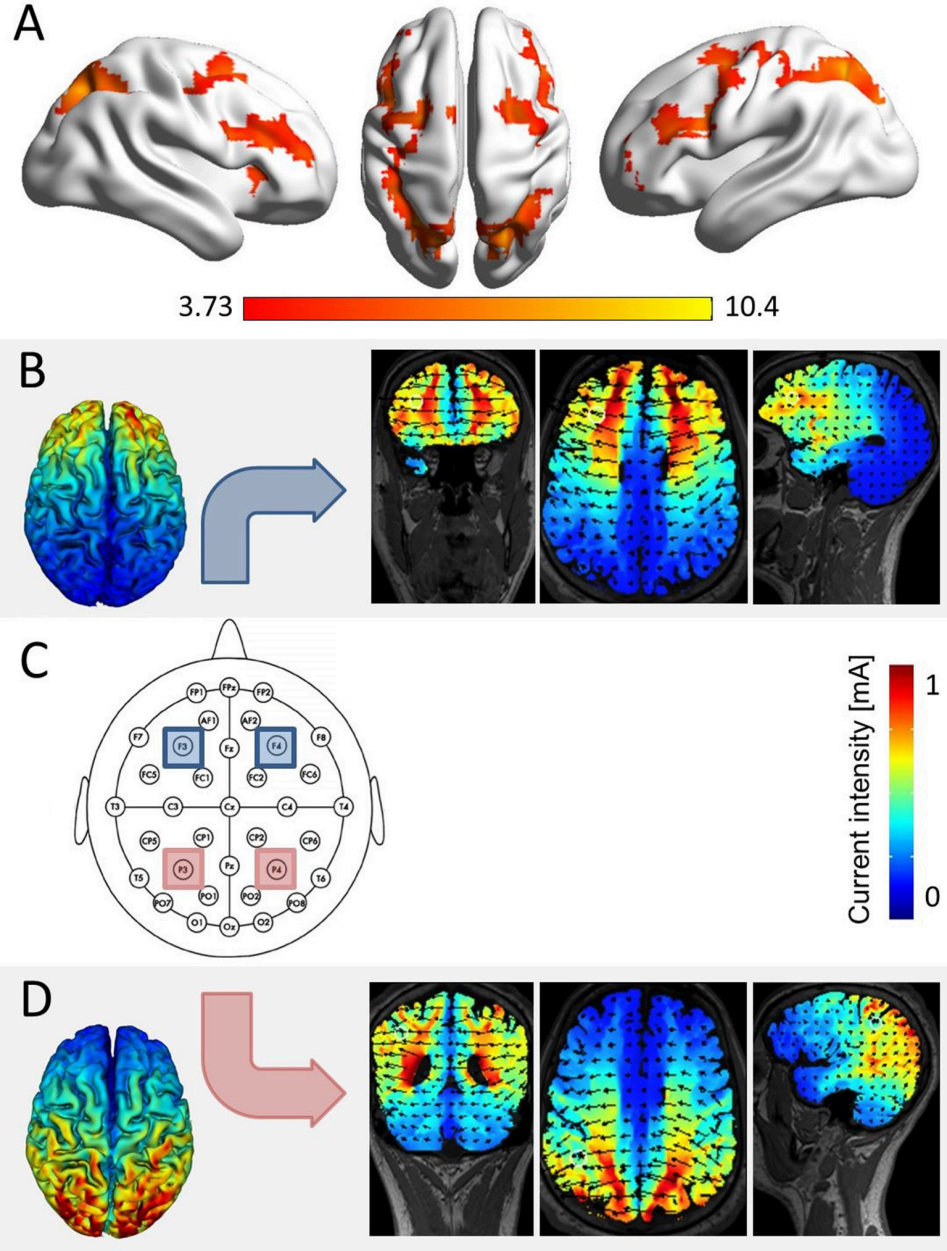
Simulation of brain areas expected to be stimulated by our stimulation protocol. **(A)** The network of joint fronto-parietal activation for the effects of distractor distance, carry, and target identity as identified in a re-analysis of fMRI data of Klein et al. (2009, conjunction analysis over the main effects of distractor distance, carry, and target identity at p_cluster−corr._ < 0.05, cluster size = 10 voxels). All three effects elicited significant bilateral parietal and frontal activation, corresponding to the location of electrodes P3 and P4 (i.e., parietal), as well as F3 and F4 (i.e., frontal, over the scalp according to the international 10–20 system for EEG electrode placement). **(C)** The actual electrode placement over bilateral parietal (red) and bilateral frontal sites (blue). **(B)** Simulation for bilateral frontal stimulation, showing electrical fields, and current intensities induced in coronar, axial, and frontal slices (using HDExplore Software, SOTERIX vs. 5.0). **(D)** Simulation for bilateral parietal stimulation using the same software.

The modulation of arithmetic processing was investigated systematically using tDCS (Klein et al., 2013; Artemenko et al., 2015) based on the same addition paradigm first employed by Klein et al. (2009). In this choice reaction paradigm, participants had to select the one solution probe (i.e., the target) from two alternatives, which was either identical with the correct result or closest to it. The alternative probe (i.e., the distractor) was manipulated as being either close or far off the correct result. Moreover, for half of the problems, a carry operation had to be performed. As such, this paradigm allowed to investigate three different effects that may possibly be modulated by tES: (i) the target identity effect, (ii) the distractor distance effect, and (iii) the carry effect. On a behavioral level, the target identity effect indicates that a target is more difficult to identify when it is not the correct result of the respective addition task, but only closer to the correct result than the distractor. Klein et al. (2013, 2016) suggested that identifying the correct result addresses processes of recognition and familiarity. This means that whenever the target is identical to the correct result, the problem may be solved by some kind of matching rather than magnitude comparison between correct result, target, and distractor. In turn, this leads to faster and more accurate responses. Second, the distractor distance effect indicates that rejecting the distractor is more difficult when the distractor is numerically close to the target solution probe (e.g., 24 + 33 = 57 or 55 vs. 24 + 33 = 57 or 43). Thereby, the distractor distance effect indicates specific numerical processing. Finally, the carry effect reflects that the respective addition problems become more difficult when a carry operation is needed. This is assumed to stem from the necessity to update the tens digit of the overall result by the tens digits of the unit sum (e.g., for 19 + 28 a carry is needed as 9 + 8 = **1**7 > 10 and thus 1 + 2 + **1** = 4, making 47 as the overall result). Therefore, the carry effect was suggested to reflect processes of place-value manipulation and integration (e.g., Nuerk et al., 2015). Finally, in an initial fMRI study, Klein et al. (2009) found the distractor distance effect to be associated with a fronto-parietal network comprising activation in the bilateral intraparietal sulci as well as left inferior, superior and middle frontal gyrus. The carry effect was associated with activation in a network including bilateral posterior intraparietal sulcus, right anterior cingulate gyrus and bilateral middle frontal gyrus, while the target identity effect was associated with activation in bilateral intraparietal sulcus and left dorsolateral prefrontal cortex. Thus, all three effects elicited activation in bilateral IPS (cf. Figure 1A). However, the fMRI methodology does not allow to distinguish whether the IPS is merely co-activated in these effects or indeed functionally necessary to solve the task. In order to evaluate such a causal structure-function relationship between the IPS and specific components of number processing in a follow-up study, tES was applied during this paradigm.

Therefore, Klein et al. (2013) investigated the impact of bilateral bi-cephalic tDCS on these effects (i.e., distractor distance, carry, and target identity effect) by applying low-intensity direct current over the IPS with two active electrodes of the same polarity. Results indicated that only the distractor distance effect was modulated by bilateral bi-cepahlic tDCS. This means that the distractor distance effect was significantly reduced under anodal as compared to cathodal stimulation (Klein et al., 2013). However, the carry effect and the target identity effect remained unaffected by tDCS. Importantly, stimulation effects were specific to number processing since stimulation did not affect a color word stroop control task. Taken together, this first stimulation study indicated that bilateral IPS specifically contributes to magnitude processing. This is in line with the general assumptions of Triple Code Model of numerical cognition (Dehaene and Cohen, 1995, 1997; Dehaene et al., 2003). Nevertheless, Klein et al. (2013) could not rule out that there might be differential contributions of left and right IPS, which could not be evaluated using bilateral bi-cephalic stimulation of the same polarity.

To address this issue, Artemenko et al. (2015) applied unilateral tDCS over the left and right IPS, respectively, using the same addition paradigm to investigate whether there was a hemispheric intraparietal specialization/difference for distractor distance, carry or target identity effects. The authors observed that only the carry effect was modulated by parietal tDCS applied to the right hemisphere. Based on these results, they concluded that the right IPS plays an important role in place-value processing (Artemenko et al., 2015). Again, no stimulation effect was found for the stroop control task. Together with the study of Klein et al. (2013), the results of this second stimulation study suggest that number magnitude processing is subserved by the bilateral IPS, while the right IPS is specifically involved in place-value processing. However, it needs to be pointed out that tES has a low spatial resolution in general and in particular so when using rectangular-pad electrode configurations compared to ring electrode configurations (e.g., Datta et al., 2009). Therefore, we will not refer to stimulated areas in the remainder of this article (e.g., we stimulated IPS) but only specify where tRNS was applied (e.g., tRNS applied over IPS).

Nevertheless, it is important to note that mental arithmetic is not only associated with the parietal cortex, but involves a wide-spread fronto-parietal network (Klein et al., 2009, 2016; Arsalidou and Taylor, 2011). So, the question remains what components of mental arithmetic are subserved specifically by frontal parts of this network. Therefore, it would be interesting to apply tES to both frontal (e.g., dorsolateral prefrontal cortex, DLPFC) and parietal parts of this network (e.g., IPS). This was pursued in the current study.

### The present study

The studies outlined above constitute a systematic investigation of the structure-function relationship of the IPS and several components of numerical cognition on the same paradigm by using fMRI (Klein et al., 2009), bilateral bi-cephalic tES (Klein et al., 2013), and unilateral bi-cephalic tES (Artemenko et al., 2015). However, so far these tES studies only evaluated effects of tDCS applied to IPS. There is currently no study investigating respective effects for tES applied to frontal areas using this established paradigm. Moreover, it might be interesting to realize a comparison of the impact of tDCS and tRNS on arithmetic processing. Therefore, the present study aimed at investigating the impact of tRNS applied to parietal and frontal areas on addition problems in terms of modulation of the distractor distance effect, the carry effect and the target identity effect by using the same paradigm and experimental procedure as in the previous studies (Klein et al., 2009, 2013; Artemenko et al., 2015). In particular, we aimed at evaluating whether parietal tRNS would modulate the distractor distance effect (Klein et al., 2013) as well as the carry effect (Artemenko et al., 2015) as observed in previous studies for tDCS. Although Klein et al. (2013) and Artemenko et al. (2015) found tDCS effects on the processing of distractor distance and the carry operation when directly comparing effects of cathodal vs. anodal stimulation, we would nevertheless expect a more pronounced effect of tRNS on the distractor distance as well as the carry effect when applying tRNS over IPS, because previous studies indicated that tRNS effects seem to be more pronounced as compared to effects of anodal tDCS (at least in motor cortex, e.g., Terney et al., 2008; Moliadze et al., 2014).

Additionally, we evaluated whether tRNS over prefrontal areas (including the DLPFC) affects arithmetic processing. As previous tRNS studies applying tRNS to frontal areas observed effects on rates of arithmetic learning but also actual task performance, we also expected such stimulation effects when frontal areas in the network of arithmetic processing are targeted (e.g., Snowball et al., 2013; Pasqualotto, 2016). However, the exact nature of these effects can hardly been predicted from previous studies using different tasks, stimuli, and procedures. As such, the evaluation of effects of frontal tRNS remains explorative.

## Methods

### Participants

Forty-eight healthy student volunteers (29 females; mean age = 23.48 years, *SD* = 3.30 years) participated in the study. Forty-five participants were right-handed as assessed by the Edinburgh Handedness Inventory (Oldfield, 1971). Moreover, participants were native German speakers and reported no history of neurological or psychiatric disorders.

All participants signed an informed consent form prior to the study and received monetary compensation or study credits for successfully completing the study. The study was approved by the local ethics committee of the Medical Faculty of the University of Tuebingen.

### Design and stimuli

The experimental design of the study was a within-subject design including two tasks (i.e., addition vs. control), and three stimulation conditions (i.e., frontal stimulation vs. parietal stimulation vs. sham). The addition task was identical to the one used by Klein et al. (2009) (see also Klein et al., 2013; Artemenko et al., 2015). For each of the three stimulation conditions, a matched stimulus set of 192 two-digit addition problems was used. Stimulus sets were identical to the ones used in Klein et al. (2013) and Artemenko et al. (2015).

In a choice reaction paradigm, addition problems were presented together with two solution probes in Arabic notation (Arial, font size 26) using white script against a black background on a 19″ screen driven at a resolution of 1,280 × 1,024 pixels. Participants had to decide which one of the two solutions was identical or closest to the correct sum by pressing a corresponding button. Stimuli were presented until a button was pressed or the time limit of 5,000 ms was reached. Responses or time outs were followed by the fixation cross (presented for 500 ms) for the next item. Each 48 trials a short break of 15 s was interposed. In the item sets the three factors distractor distance (small vs. large), carry (without vs. with carry), and target identity (non-identical vs. identical) were manipulated orthogonally. For details see Klein et al. (2009). Prior to the testing phase with the critical items, participants completed a practice phase with 32 trials. Trial order of the addition task was randomized. Overall, the task lasted about 17 min.

A color word stroop task was used as a control task. Color words were presented in different colors (e.g., the word “RED” written in blue color) at the center of a black screen. Participants were instructed to identify the written color of the presented word and press a corresponding button. Stimuli were presented until a button was pressed or the time limit of 2,000 ms was reached. At the beginning of each trial, a fixation cross was presented for 300 ms followed by a blank screen for 500 ms. The control stroop task consisted of 24 practice trials followed by 96 critical trials, for which trial order was randomized. The control task lasted about 3 min.

### tRNS application

tRNS was conducted while participants performed the task. Stimulation was applied either to the bilateral IPS (parietal stimulation), the bilateral DLPFC (frontal stimulation), or the stimulation protocol followed a sham procedure (sham condition). To blind participants for the stimulation condition in each session, all four electrodes covered with saline-soaked synthetic sponges (each with a size of 5 × 5 cm^2^) were placed over the areas P3/P4 (corresponding to the IPS) and F3/F4 (corresponding to the DLPFC; see Figure 1C) according to the international 10–20 system for EEG electrode placement (Jasper, 1958; Okamoto et al., 2004) in each of the three testing sessions. During tRNS, only parietal or frontal electrodes were active in the stimulation conditions.

Stimulation was delivered by a multichannel DC Brain Stimulator device (DC-Stimulator MC, neuroConn, Illmenau, Germany). A 1 mA (range from −0.5 to 0.5 mA) high frequency (100–640 Hz) random noise stimulation was applied to the target regions as it is considered to trigger neural excitation more strongly than lower frequency stimulation (see also Terney et al., 2008). For both stimulation conditions, current was applied for a duration of 20 min, with a ramp-up and ramp-down phase of 15 s, respectively. A simulation study prior to the experiment indicated that with the chosen placement of electrodes we induced current flow in the respective target areas (i.e., IPS and DLPFC, see Figures 1B,C). In the sham condition, current was applied for 30 s (with additional ramp-up and ramp-down phases of 15 s).

### Procedure

The experimental procedure was similar to the one of Klein et al. (2013). In a within-subject design, each participant underwent all three stimulation conditions (i.e., frontal, parietal, and sham) in three sessions. The order of stimulation conditions was counterbalanced across participants. It was ensured that a minimum interval of 6 days (*M* = 7.17; *SD* = 0.65) separated sessions to avoid short-term training effects and long-term stimulation effects (see Cohen Kadosh et al., 2010). In each session, participants completed the addition task prior to the control stroop task. To ensure that stimulation effects would establish accurately, tRNS started simultaneously with the training phase (see Nitsche et al., 2008) and the testing phase was started 5 min after stimulation onset. Stimulation was terminated after 20 min. Moreover, to minimize learning effects different stimulus sets were used for each session and counterbalanced over all participants. Overall, each session lasted about 60 min.

### Data analysis

Analyses of reaction times (RT) of the addition task and the control stroop task were performed using R (R Development Core Team, 2016) and SPSS (Version 22.0). Practice trials were not considered in the analyses. RT analyses were based on correct trials only resulting in a loss of 14.58% of the data for the addition and 4.79% for the stroop task. Furthermore, response latencies smaller than 200 ms were not considered, and in a second step responses outside the interval of ±3 standard deviations around the individual mean were excluded. An additional 0.72% and 0.02% of the data was excluded due to this trimming procedure for the addition and stroop task, respectively. For the addition task, a 3 × 2 × 2 × 2 ANOVA was conducted discerning the factors stimulation (parietal stimulation vs. frontal stimulation vs. sham), distractor distance (small vs. large), carry (without vs. with carry), and target identity (non-identical vs. identical). Moreover, for the control stroop task a 3 × 2 ANOVA with the factors stimulation (parietal stimulation vs. frontal stimulation vs. sham) and congruency (congruent vs. incongruent) was conducted.

## Results

### Addition task

The ANOVA revealed reliable main effects of distractor distance [*F*_(1, 47)_ = 24.92, *p* < 0.001], carry [*F*_(1, 47)_ = 164.21, *p* < 0.001] and target identity [*F*_(1, 47)_ = 58.56, *p* < 0.001]. Participants responded faster to large compared to small distractor distances (2,672 ms vs. 2,746 ms, respectively), to non-carry as opposed to carry problems (2,537 ms vs. 2,880 ms, respectively), and to identical compared to non-identical targets (2,636 ms vs. 2,782 ms, respectively). Moreover, there was a significant interaction of distractor distance and target identity [*F*_(1, 47)_ = 12.71, *p* < 0.001] and of carry and target identity [*F*_(1, 47)_ = 9.79, *p* < 0.005]. This indicated that the distractor distance effect was larger for non-identical targets than for identical targets (100 ms vs. 49 ms, respectively), and the carry effect was larger for identical targets than for non-identical targets (373 ms vs. 311 ms). These results replicate the findings of previous studies by Klein et al. (2009, 2013) and Artemenko et al. (2015). Additionally, there was a three-way-interaction of distractor distance, carry, and target identity [*F*_(1, 47)_ = 8.29, *p* < 0.01]. Breaking down this three-way interaction into two two-way interactions revealed that the interaction between distractor distance and carry was significant for identical targets [*F*_(1, 47)_ = 17.15, *p* < 0.001] but not for non-identical targets [*F*_(1, 47)_ < 1, *p* = 0.595]. For identical targets this indicated that the distractor distance effect was more pronounced for non-carry as compared to carry problems (69 ms vs. 5 ms).

**Figure 2:**
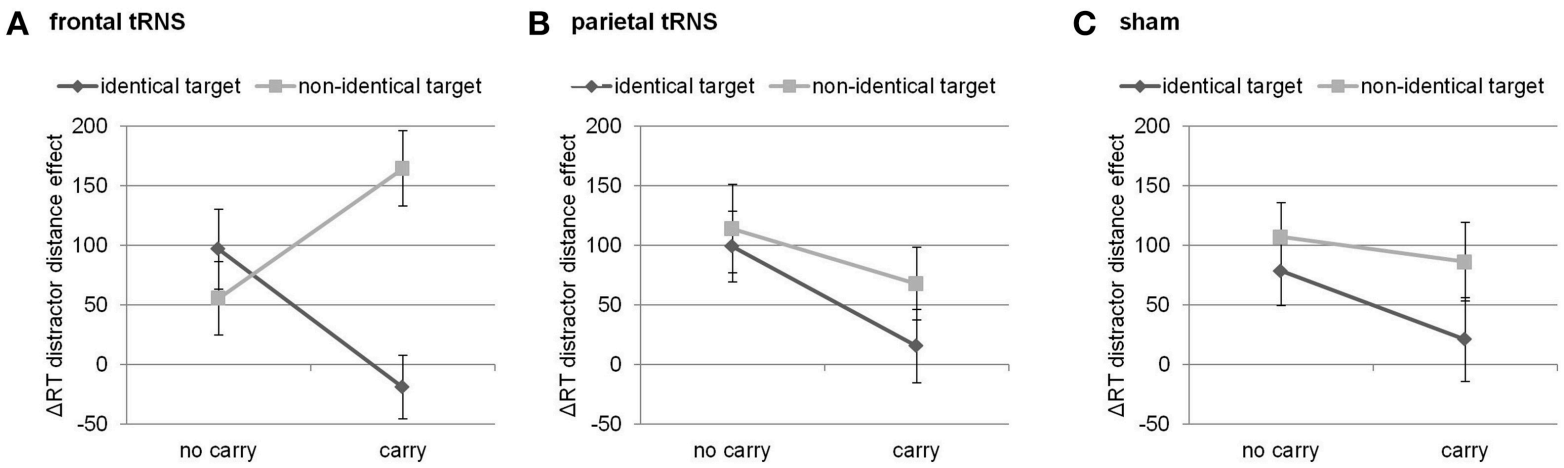
Three way-interaction of distractor distance effect, carry, and target identity for **(A)** frontal stimulation, **(B)** parietal stimulation, and **(C)** sham. Only for frontal stimulation the interaction of carry and target identity was significant. Error bars indicate standard errors.

Finally, this three-way interaction was qualified by the four-way interaction of distractor distance, carry, target identity, and stimulation [*F*_(2, 94)_ = 3.49, *p* < 0.05]. Breaking down this four-way-interaction into its constituting three-way interactions revealed that the three-way interaction between distractor distance, carry, and target identity was only significant for frontal stimulation [*F*_(1, 47)_ = 15.40, *p* < 0.001], but not for parietal [*F*_(1, 47)_ < 1, *p* = 0.549] nor sham stimulation [*F*_(1, 47)_ < 1, *p* = 0.523, see Figure 2]. Further breaking down this three-way interaction for frontal stimulation into its constituting two-way interactions indicated that the interaction of distractor distance and carry was significant for both identical [*F*_(1, 47)_ = 6.73, *p* < 0.05] as well as non-identical targets [*F*_(1, 47)_ = 7.45, *p* < 0.01]. However, considering the marginal means revealed opposing influences of carry on the distractor distance effect for identical and non-identical targets. For non-identical targets, the distractor distance effect was more pronounced for carry as compared to non-carry problems (165 ms vs. 56 ms, see Figure 2) whereas this was reversed for identical targets with a larger distractor distance effect for non-carry as compared to carry problems (97 ms vs. −19 ms). In particular, the distractor distance effect for identical targets with carry was significantly different from the distractor distance effect for non-identical targets with carry [*t*_(47)_ = −4.62, *p* < 0.001], while the difference between the distractor distance effects for identical and non-identical targets without carry remained insignificant [*t*_(47)_ = 1.01, *p* = 0.320].

All other main effects and interactions were not significant (all *Fs* ≤ 2.82, all *ps* > 0.10). Importantly, there was no significant interaction with stimulation with the exception of the above described four-way interaction. Additionally, we calculated the mean distractor distance effect for the three stimulation conditions sham, frontal and parietal stimulation (2,756–2,690 ms = 66 ms vs. 2,765–2,696 ms = 69 ms vs. 2,753–2,676 ms = 77 ms, respectively).

As we did not observe an interaction of distractor distance effect and stimulation, we performed null effect testing using the Bayesian method (cf. Masson, 2011). Here, the Bayesian analysis revealed positive evidence in favor of the null hypothesis (*BF*_01_ = 6.91), this means no modulation of the distractor distance effect through parietal stimulation. Furthermore, we also did not find an interaction of carry effect and stimulation. Again, Bayesian analysis revealed positive evidence in favor of the null hypothesis (*BF*_01_ = 6.72), this means no modulation of the carry effect through parietal stimulation.

### Stroop task

The ANOVA revealed a significant main effect of stroop congruency [*F*_(1, 47)_ = 88.09, *p* < 0.001], indicating faster responses for congruent than for incongruent trials (640 vs. 701 ms). The main effect of stimulation as well as its interaction with congruency did not reach significance (all *Fs* ≤ 0.71, *ps* > 0.48).

## General discussion

In this study, we evaluated influences of tRNS over the bilateral IPS or bilateral DLPFC on the effects of distractor distance, carry, and target identity while participants performed an addition task. As previous studies indicated that applying tRNS led to more pronounced and longer lasting effects than tDCS (at least in the motor cortex, e.g., Terney et al., 2008; Moliadze et al., 2014), we were interested whether we would observe a more pronounced modulation of the distractor distance effect and carry effect by means of tRNS in the current study. Furthermore, as previous studies also showed strong influences of tRNS applied over prefrontal areas (i.e., including the DLPFC) on numerical cognition, we also aimed at investigating the influence of frontal tRNS during mental addition on the effects of distractor distance, carry, and target identity.

All behavioral effects (i.e., distractor distance effect, carry effect, target identity effect, and their interactions) as found in previous studies (Klein et al., 2009, 2013; Artemenko et al., 2015) were replicated in this study indicating that our experimental setup was efficient. Nevertheless, a modulation of the distractor distance and carry effect by parietal tRNS was not observed, although compared to previous studies (Klein et al., 2013; Artemenko et al., 2015) we doubled our sample size. Bayesian analysis of the parietal modulation of the distractor distance as well as the carry effect revealed substantial evidence in favor of the null hypothesis that parietal stimulation did not modulate these effects in the current study. Nevertheless, we observed spurious effects as shown by a four-way interaction effect indicating possible influences of frontal tRNS on the distractor distance effect (which we will discuss in more detail below).

In line with previous tDCS studies (Klein et al., 2013; Artemenko et al., 2015), no stimulation effects in the control stroop task were found. In the following, the meaning of our tRNS findings compared to the findings of these previous tDCS studies will be discussed.

### No modulation of the distractor distance and carry effect during parietal stimulation

In contrast to our expectations, we did not find a modulation of the distractor distance effect by parietal tRNS as substantiated by Bayesian null effect testing. Because the effect of distractor distance reflects number magnitude processing (Klein et al., 2016), our results seem to suggest that parietal tRNS did not influence the processing of number magnitude information as bilateral parietal tDCS did (Klein et al., 2013). Similarly, we did not observe a stimulation effect on the carry effect as reported for unilateral tDCS by Artemenko et al. (2015). This indicates that place-value manipulation also seemed to be unaffected by tRNS in our study.

This lack of evidence for tRNS effects was unexpected; in particular, as we doubled the sample size to 48 participants as compared to previous studies (*n* = 24 in Klein et al., 2013; *n* = 25 in Artemenko et al., 2015) to ensure sufficient power to detect small stimulation effects. While the larger sample size might still not fully exclude power issues, the use of an identical arithmetic paradigm and a similar stimulation setting at any rate allows for a comparison of the effect sizes. Therefore, the lack of modulation of the distractor distance effect and the carry effect by parietal tRNS suggests that—at least in our task with an identical experimental setting—effects of parietal tDCS on the distractor distance effect (in case of bilateral bi-cephalic stimulation) and on the carry effect (in case of unilateral bi-cephalic stimulation) seemed to be stronger than effects of parietal tRNS. In turn, tRNS does not seem to be more effective than tDCS in general as put forward by previous studies using tRNS (e.g., Terney et al., 2008; Moliadze et al., 2014).

Even though our simulation prior to the experiment indicated significant current flow in the respective parietal and frontal target areas (cf. Figure 1), a possible explanation for the inconsistency of our findings with the literature may lie in neuro-anatomical topography. This means that tRNS was applied over a gyrus such as the motor cortex in previous studies (Terney et al., 2008; Moliadze et al., 2014) in comparison to tRNS applied over a sulcus such as the IPS in the current one. The motor cortex is situated in the precentral gyrus, which means neural tissue located directly under the scalp and thus possibly more exposed to current flow applied over the scalp. In contrast, it is assumed that the crucial sites for numerical processing are located in the fundus of the IPS (e.g., Dehaene et al., 2003), an anatomical structure which is typically at least about 2 cm from the cortex surface (e.g., Caspers et al., 2006). However, higher effectiveness of tRNS as compared to tDCS was also shown for other cortex areas (e.g., the visual cortex, Fertonani et al., 2011).

Similar to the motor cortex, significant parts of the DLPFC such as the middle frontal gyrus are also situated directly below the scalp. The middle frontal gyrus was found highly activated for all three effects when re-analyzing the fMRI data from Klein et al. (2009, see Figure 1A). Interestingly, we observed a significant three-way interaction of the effects observed within frontal tRNS. While an interpretation of this interaction has to remain highly speculative (see below for a tentative account), this finding is in principal accordance with the idea of possible anatomical constraints for tRNS.

Nevertheless, it has to be noted that previous studies of Klein et al. (2013) and Artemenko et al. (2015) were able to demonstrate effects for bilateral as well as unilateral tDCS stimulation over the parietal cortex within exactly the same experimental paradigm. Therefore, in our paradigm effects of tDCS seemed to be more pronounced than effects of tRNS, independent of parietal anatomy. One possible explanation for this lack of modulation by parietal tRNS might be that tRNS compared to tDCS relies on different neurophysiological mechanisms. The exact working mechanism is so far not known. However, it has been argued that applying randomly alternating currents adds neural “white” noise (Terney et al., 2008). In turn, the increase of background noise is assumed to boost the neural signal toward the activation threshold (by means of repetitive opening of Natrium channels of neurons, e.g., Antal and Herrmann, 2016). Animal studies are needed to provide further insights into the physiological mechanisms underlying tRNS.

Another explanation might be the different orientation of field lines in the tDCS studies (Klein et al., 2013; Artemenko et al., 2015) compared to the present study as indicated by the respective simulations. In both tDCS studies, field lines (and thus current flow) were oriented from anterior to posterior, whereas in the current tRNS study the orientation of field lines were oriented from left to right hemisphere. This might have influenced neural populations differently leading to the observed different stimulation effects on the distractor distance and the carry effect.

Furthermore, also the type of study might be an important point which needs to be mentioned: almost all other studies on numerical cognition, which apply tRNS through frontal and parietal electrodes, are intervention studies to enhance numerical learning (Cappelletti et al., 2013; Snowball et al., 2013; Popescu et al., 2016; Looi et al., 2017; but see Pasqualotto, 2016 for an exception). Therefore, tRNS might simply be more beneficial in enhancing numerical learning than in modulating actual performance on a numerical task. Importantly, this argument is in line with the results of a recent meta-analysis by Simonsmeier et al. (2018) that showed that effect sizes of tES applied were generally larger when applied during a learning phase (*d* = 0.712) as compared to its application during a test phase (*d* = 0.207).

Finally, there is evidence showing that factors such as stimulation intensity, cognitive state and task difficulty modulate the impact of brain stimulation on behavior (cf. Sandrini et al., 2011; de Graaf et al., 2014; Romei et al., 2016 for reviews on TMS). In their theoretical model, Silvanto and Cattaneo (2017) suggest the effect of stimulation intensity to be highly dependent on neural excitability, which is determined by cognitive state. The authors argue that an intensity which, for instance, typically induces suppression can have a facilitatory effect in case stimulated neurons are already inhibited by ongoing task-related processes (i.e., the actual cognitive state) or the other way around.

Accordingly, the impact of stimulation seems dependent on the initial cognitive state to which the stimulation is applied: brain stimulation (e.g., by tRNS) was argued to influence actual brain activity differently when participants perform a specific task before stimulation as compared to when participants start performing a task during stimulation (e.g., Silvanto et al., 2008; for reviews see also Rudiak and Marg, 1994; Romei et al., 2016). When stimulation starts during an ongoing task, the relevant neurons may already have adapted to the task at hand, so that they are more likely in a stable state of excitability. In turn, this should reduce variability of stimulation effects. Because this applies to both, the present tRNS study as well as to previous tDCS studies (Klein et al., 2013; Artemenko et al., 2015), future studies are needed in which state dependency of the respective stimulation effects should be investigated.

Taken together, application of parietal tRNS during two-digit addition seemed to be less effective than parietal tDCS. Studies that reported larger tRNS modulation effects either employed different cognitive tasks (i.e., learning paradigms instead of arithmetic testing only) or stimulated different locations, which may be more exposed to current flow (i.e., precentral gyrus instead of the deeper intraparietal sulcus). Nevertheless, in contrast to parietal tRNS, we found an effect of frontal tRNS on the interaction of all numerical factors. In the following, some tentative interpretation as to what this might imply is sketched.

### Modulation of mental arithmetic by frontal stimulation

Our results indicated that tRNS over frontal cortices as opposed to sham or parietal stimulation seemed to modulate the interaction of distractor distance, carry, and target identity. Breaking down this three-way interaction indicated that this can be interpreted as a modification of the distractor distance effect. In particular, during frontal tRNS the distractor distance effect was largest in the easiest (i.e., identical targets without a carry) as well as the hardest condition (i.e., non-identical targets with carry), so to speak the “most extreme” conditions. Additionally, for the other conditions (i.e., non-identical targets without a carry and identical targets with carry) the distractor distance effect was smaller (i.e., mixed conditions). This indicates that for these extreme conditions the distractor plays a more prominent role because the effect of distractor distance increases during frontal stimulation.

In the behaviorally easiest condition (i.e., identical targets without a carry), this may indicate that target recognition might be facilitated as the target is identical to the correct result of the respective addition and no carry is needed. Therefore, the identical target should be identified most easily. In the neuroimaging data on the same paradigm, an identical effect was observed for the activation in areas related to the recognition of familiar objects (Klein et al., 2016). However, if distractor distance decreases, participants most probably had to additionally evaluate and reject the distractor. This additional processing and evaluating of the distractor takes more time, which is also reflected by increasing distractor distance effect.

When applied to the behaviorally most difficult condition (i.e., non-identical targets with carry) both of these short-cut strategies, which aim at avoiding to actually calculate the final result, may be detrimental as it is not possible to recognize the target as the correct result of the addition problem. Instead, the number closest to the correct result thus had to be chosen. In this case, decreasing distractor distance should be specifically detrimental as it makes the differentiation between the target (which nevertheless differs from the correct result of the addition problem) and the distractor and thus rejecting the distractor particularly more difficult. Therefore, it is necessary to consider two different distances simultaneously (i.e., distractor distance and the distance between the target and the correct result). In turn, this additional necessity to process number magnitude information may lead to a stronger distractor distance effect.

In the two other conditions (i.e., identical targets with a carry and non-identical targets without a carry) it is most reasonable to calculate since short-cut strategies such as matching the target or rejecting the distractor may not be as easy to accomplish (e.g., as the carry has to be considered when trying to estimate the tens digit of possible results from that of the summands). Therefore, the distractor may be considered less helpful in solving such addition problems and the distractor distance effect becomes smaller during frontal tRNS.

As such, these differential patterns of results for the distractor distance effect indicate that frontal stimulation, in particular the stimulation of the DLPFC, may influence the choice processing strategies instead of directly influencing the processing of number magnitude information: depending on the degree of difficulty, the tendency to refer to short-cut strategies avoiding or complementing actual calculation procedures on the distractor may be more (i.e., easiest and hardest condition) or less (i.e., mixed conditions) pronounced. Nevertheless, due to the complex nature of this interaction, this approach on the effect of frontal tRNS has to remain putative and speculative until substantiated by future research.

### Modulation of specific effects vs. general modulation via neuro-stimulation

In line with previous studies (Klein et al., 2013; Artemenko et al., 2015) we did not find an effect of tES on the control stroop task. However, as the stroop task involves processes of cognitive control (e.g., Egner and Hirsch, 2005), one may have expected stimulation effects during DLPFC stimulation; in particular, as DLPFC is an area associated with cognitive control (MacDonald et al., 2000). The lack of modulation of the stroop effect through DLPFC stimulation might be explained by the large neural network associated with cognitive control. Not only DLPFC has been associated with cognitive control, but also anterior cingulate cortex (ACC). In the context of the stroop task, MacDonald et al. (2000) found that left DLPFC seemed to be more active for color naming than for word reading (i.e., executing inhibitory control) whereas ACC was more active when responding to incongruent stimuli (i.e., performance monitoring). Therefore, not only DLPFC may be involved but also, amongst others, ACC—a brain region which was not affected by DLPFC stimulation (cf. Figure 1B). Still, the lack of stimulation effects on performance in the stroop control task indicates that stimulation effects in this study (but also in previous studies, i.e., Klein et al., 2013; Artemenko et al., 2015) were specific to number processing.

While these studies showed stimulation effects on specific numerical *effects* (Klein et al., 2013; Artemenko et al., 2015), other studies found stimulation effects on performance in numerical *tasks* more generally (e.g., Hauser et al., 2013; for a differentiation of effect and task approach see Moeller et al., 2011). In this stimulation study, Hauser et al. (2013) found a general performance improvement in a subtraction and a number comparison task during anodal tDCS applied to left posterior parietal cortex. Therefore, it is still not clear which stimulus protocols and tasks enhance general performance and which affect specific components of numerical processing. More studies are needed to disentangle stimulation effects on specific effects and/or numerical tasks more generally (cf. Moeller et al., 2011 for a discussion).

## Conclusion

In the current study, we used an established two-digit addition task and experimental setting to evaluate the effects of parietal and frontal tRNS on specific numerical effects. Previous tDCS studies reported a modulation of the distractor distance and carry effect (reflecting number magnitude and place-value processing) by parietal stimulation in this task. In contrast, however, we did not find any effect of parietal tRNS on two-digit addition performance. As such, our findings suggest that tRNS application to parietal cortex sites during mental arithmetic seems to be less effective than parietal tDCS. As a small and specific effect we found a modulation of the distractor distance effect by frontal tRNS—however, only for the extreme conditions (i.e., most easiest and most difficult conditions; identical targets without a carry and non-identical targets with carry). In sum, we suggest that tRNS application during the actual performing of numerical testing tasks (as compared to learning phases) may be less effective than tDCS—at least when applied over the IPS. This is in line with the results of a recent meta-analysis (Simonsmeier et al., 2018), which found that tES application in learning paradigms might be more effective. Future studies are needed to identify whether or not effects of tRNS in learning paradigms are actually stronger than effects of tDCS.

## Author contributions

SB, CA, and EK: Designed the study; SB: Conducted the study; SB, EK, and KM: Analyzed the data; SB, CA, KM, and EK: Wrote the manuscript.

### Conflict of interest statement

The authors declare that the research was conducted in the absence of any commercial or financial relationships that could be construed as a potential conflict of interest.
